# Multi-UAV Cooperative Hunting in Obstructed Environments via a Multi-Agent Proximal Policy Optimization with Curriculum Learning

**DOI:** 10.3390/s26123907

**Published:** 2026-06-19

**Authors:** Longjie Zheng, Junlin Zhou, Haijun Peng, Bai Li, Xinwei Wang

**Affiliations:** 1 Department of Engineering Mechanics, State Key Laboratory of Structural Analysis, Optimization and CAE Software for Industrial Equipment, Dalian University of Technology, Dalian 116024, China; 1563162495@mail.dlut.edu.cn (L.Z.); hjpeng@dlut.edu.cn (H.P.); 2 School of Mathematical Science, Dalian University of Technology, Dalian 116024, China; zhoujunlin@mail.dlut.com; 3 School of Information & Electronic Engineering, East China Normal University, Shanghai 200241, China; libai@zju.edu.cn

**Keywords:** UAV, cooperative hunting, noncooperative target, CL-MAPPO, obstructed environment

## Abstract

With the increasing complexity of unmanned aerial vehicle (UAV) missions in complex obstacle environments, cooperative hunting of maneuvering ground targets by UAV swarms has become an important problem for multi-agent autonomous decision-making. This paper focuses on a simulated three-UAV hunting scenario in a two-dimensional obstructed environment, where UAVs must search for, approach, encircle, and continuously track a target while avoiding static obstacles under local observation. To address the problem of multi-UAV cooperative hunting of dynamic targets in complex obstacle environments, this paper proposes a curriculum learning (CL)-based Multi-Agent Proximal Policy Optimization algorithm, termed CL-MAPPO. Specifically, a three-stage progressive training curriculum is designed to overcome the challenges of low exploration efficiency, slow environmental adaptation, and difficult convergence of cooperative hunting policies faced by multi-agent deep reinforcement learning in hunting tasks, thereby gradually enhancing the cooperative hunting capability of UAVs in complex environments. Curriculum I employs fixed obstacles and a stationary target position to train the UAVs’ basic obstacle avoidance and target search abilities. Curriculum II introduces randomly generated obstacles and target positions to improve the UAVs’ adaptability to varying environments. Curriculum III further incorporates a dynamic target, prompting the UAVs to learn effective hunting strategies against maneuvering targets. The simulation experiment includes ablation experiments against MAPPO without curriculum learning and comparative simulations against MADDPG and MADQN, using reward convergence curves and trajectory visualizations to evaluate the training results. The results show that, under the same training episodes in the ablation experiment, CL-MAPPO reaches a higher and more stable reward level than vanilla MAPPO, indicating improved learning efficiency without increasing model complexity. In the comparative experiment, the CL-MAPPO algorithm achieved a higher success rate in cooperative hunting. These simulation experiments verify the effectiveness and superiority of the CL-MAPPO algorithm in multi-agent cooperative hunting tasks.

## 1. Introduction

### 1.1. Background and Significance

With the rapid development of artificial intelligence and UAV technologies, the application of UAVs has become increasingly widespread in various fields [1,2,3,4]. For multi-agent cooperative hunting within complex environments, UAV swarms play a pivotal role by collaboratively searching, encircling, and capturing dynamic targets, thereby significantly improving mission execution efficiency. Nevertheless, the high speed and agility of UAVs, coupled with the dynamic and obstacle-rich environment, pose severe challenges for human operators, who often struggle with timely and accurate decision-making under intense cognitive load. Meanwhile, traditional decision-making approaches, such as rule-based strategies, artificial potential field (APF) methods, and optimization-based algorithms, often suffer from local minima and high computational complexity [5,6]. Similarly, finite state machines (FSM) [7] suffer from the state explosion problem and lack the reactivity required for dynamic environments, while heuristic search algorithms [8,9] struggle with real-time performance and adaptability as they heavily rely on global map information.

Consequently, there is a pressing need to investigate real-time intelligent decision-making methods for multi-agent cooperative hunting in complex environments. Deep reinforcement learning (DRL), with its capability to learn sophisticated strategies directly from high-dimensional inputs, offers a promising solution [10,11,12]. Integrating CL further enables agents to progressively master increasingly challenging tasks, improving both learning efficiency and policy robustness. Research on multi-agent cooperative hunting not only advances the application of UAV technology but also establishes a foundation for future intelligent and networked unmanned systems. To this end, this paper proposes a CL-MAPPO to address the cooperative hunting of noncooperative maneuvering targets in complex obstacle environments.

### 1.2. Related Works

#### 1.2.1. Deep Reinforcement Learning for Multi-Agent Decision-Making

Multi-agent deep reinforcement learning (MADRL) provides a powerful framework for addressing sequential decision-making problems in cooperative hunting tasks, and existing methods can be broadly categorized into value-based and policy-based approaches.

Value-based MADRL methods extend single-agent value-based reinforcement learning to multi-agent scenarios, with the core idea being to learn the optimal joint action-value function and then derive each agent’s optimal policy indirectly. To mitigate the combinatorial explosion of the joint action space, these methods decompose the joint Q-function into individual local Q-functions while leveraging centralized value networks to enhance cooperative performance. Representative algorithms include Value-Decomposition Networks (VDN) and QMIX [13,14]. VDN decomposes the joint action-value function additively into the sum of per-agent local action-value functions, i.e., $Q_{jont}\left(\tau ,a\right)=\sum_{i}Q_{i}\left(\tau_{i},a_{i}\right)$, where $\tau_{i}$ denotes the historical observation trajectory of agent *i*. This simple linear structure facilitates efficient training, but the additive assumption is overly restrictive and cannot capture complex cooperative relationships among agents, limiting its performance in challenging coordination tasks. QMIX extends VDN by introducing a mixing network that nonlinearly combines local Q-values into the joint Q-value while enforcing monotonicity constraints. This design ensures that improvements in local Q-values lead to improvements in the joint Q-value, enabling QMIX to better accommodate complex multi-agent cooperative requirements.

Policy-based MADRL methods directly model and optimize the joint policy of multiple agents, making them particularly well-suited for continuous action spaces and large-scale cooperative scenarios. Representative algorithms include MAPPO and Multi-Agent Deep Deterministic Policy Gradient (MADDPG) [10]. MAPPO adopts centralized training, decentralized Execution (CTDE) architecture [10]. Each agent maintains an independent policy network that generates actions using only local observations, while a centralized critic network utilizes global environmental information to evaluate the value of the joint policy, computing advantage functions to guide the policy updates of all agents. This architecture is characterized by simple implementation, training stability, and strong scalability, effectively mitigating the non-stationarity problem in multi-agent environments. In contrast, MADDPG equips each agent with both an independent policy network and a centralized critic network. The policy network generates actions based solely on local observations, whereas the critic network can access the observations and actions of all agents to evaluate the value of joint actions, providing each agent with more accurate gradient information for policy improvement. This architecture has demonstrated strong performance in continuous control, cooperative navigation, and multi-agent dynamic interaction scenarios.

#### 1.2.2. Development Status of Multi-Agent Cooperative Hunting

The problem of multi-agent cooperative hunting has been extensively investigated as a fundamental paradigm of distributed collaborative decision-making and motion control in multi-agent systems. The core objective is to achieve target encirclement, tracking, and stable hunting in dynamic and complex environments through information interaction and behavioral coordination among multiple autonomous agents. With the rapid advancement of unmanned systems and intelligent decision-making technologies, cooperative hunting methodologies are progressively evolving from traditional control strategies toward intelligent, distributed learning frameworks, forming a technological landscape centered on reinforcement learning, attention mechanisms, artificial potential fields, and distributed control [15].

The integration of attention mechanisms with reinforcement learning constitutes a pivotal pathway for enhancing the intelligent decision-making capabilities of cooperative hunting systems, and various improved algorithms have been effectively deployed across different robotic platforms. Yu et al. [11] proposed a hybrid attention-oriented experience replay method integrated with MADDPG, which enables agents to focus on critical information during the interaction process by designing a hybrid attention module that separately encodes states and actions. This approach significantly improves experience utilization efficiency and learning convergence performance. Xue et al. [16] combined multi-head self-attention mechanisms with multi-agent reinforcement learning to further strengthen the system’s perception of target and environmental features, effectively accelerating the convergence speed of hunting policies and improving the overall task success rate in multi-unmanned surface vehicles (USV) scenarios. Wang et al. [17] introduced a knowledge-guided reinforcement learning method incorporating APF-based demonstrations, which enhances learning stability and decision quality through expert trajectory pre-training and real-time potential field guidance during the fine-tuning phase. This method has been successfully applied to multi-autonomous underwater vehicles (AUVs) cooperative hunting tasks. Wang et al. [18] developed a MAPPD approach with multi-head attention, effectively mitigating the challenges posed by sparse rewards and improving algorithmic adaptability in multi-evader environments. Furthermore, distributed algorithms based on decentralized Partially Observable Markov Decision Processes have achieved centralized training with decentralized execution, thereby enhancing the cooperative hunting capability of large-scale agent swarms [19]. Zhong et al. [20] proposed a cooperative hunting method based on advantage decomposition and sequential decision-making, enabling each agent to independently make decisions according to preceding individual behaviors and current environmental information, which further improves the stability and execution efficiency of the hunting process.

The robustness and efficiency of cooperative hunting systems can be optimized through diversified control strategies and coordination mechanisms tailored to the demands of different complex scenarios. Luo et al. [21] proposed a hybrid control strategy combining artificial rules with self-learning, which employs prior rules to realize basic hunting behaviors and subsequently utilizes the Twin Delayed Deep Deterministic Policy Gradient (TD3) algorithm for adaptive optimization. This hybrid paradigm enhances the system’s capability for tracking and encircling dynamic targets with stronger robustness. He et al. [22] developed a dynamic multi-target self-organization hunting control method for multi-agent systems based on fuzzy inference, particle swarm optimization, and potential field theory. This approach decomposes the multi-target hunting problem into multiple dynamic single-target sub-tasks, achieving efficient matching and cooperative hunting between agents and targets. Xu et al. [23] proposed a two-stage distributed control algorithm to address the moving target surrounding control problem under input saturation constraints, driving multi-agent systems to stably encircle dynamic targets in uniform formations. To reduce unnecessary information transmission while maintaining hunting performance, Xu et al. [24] further developed an event-triggered surrounding a formation control method for multiple dynamic targets, effectively reducing inter-agent communication overhead. Li et al. [25] investigated consensus tracking control over asynchronous cooperation-competition networks, providing theoretical support for the complex individual interaction mechanisms inherent in the cooperative hunting process.

Nevertheless, despite the extensive research efforts devoted to multi-agent cooperative hunting in recent years, MADRL-based approaches still face significant challenges, including low exploration efficiency, slow environmental adaptation, difficult convergence of cooperative policies, and high sample complexity when dealing with continuous control in high-dimensional state-action spaces [26,27].

Classical and hybrid approaches for pursuit-evasion, formation control, and obstacle avoidance are highly relevant to the cooperative hunting problem. Pursuit-evasion games have been analyzed using differential game theory and geometric methods, but these often assume perfect information and simple environments. Formation control strategies, such as leader-follower, virtual structure, and behavior-based methods, provide effective ways to maintain desired inter-UAV geometries. However, they typically rely on accurate communication and predefined patterns. Obstacle avoidance is commonly addressed via APF [5], rapidly exploring random trees (RRT), or model predictive control (MPC). Despite their effectiveness in structured settings, these traditional methods struggle with dynamic, partially observable, and obstacle-rich environments. Hybrid approaches, e.g., combining APF with reinforcement learning [17,21], have shown promise by leveraging prior knowledge to guide exploration.

To overcome the remaining challenges, including low exploration efficiency, slow environmental adaptation, and difficult convergence in complex multi-agent tasks, CL has been introduced as a progressive training strategy. CL decomposes a complex task into a sequence of sub-tasks with increasing difficulty, guiding agents to gradually acquire sophisticated behavioral patterns [28,29]. Originally developed for supervised learning, CL has been extended to deep reinforcement learning to address sparse reward and hard exploration problems. In multi-agent settings, CL helps alleviate non-stationarity by allowing agents to master simple cooperative patterns before facing challenging interactions. Several works have applied CL to multi-agent tasks. QU et al. [28] proposed an inheritance training method based on CL for multi-agent soft actor-critic (MASAC) in USV collaboration. Li et al. [29] developed an RNN-enhanced curriculum-driven algorithm for POMDPs with limited experience. Despite these advances, the application of CL to multi-UAV cooperative hunting in obstructed environments with dynamic targets remains underexplored.

#### 1.2.3. Research Gap and Motivation

Existing multi-agent cooperative hunting methods face several critical limitations when applied to obstructed environments with maneuvering targets: (1) Most MADRL algorithms (e.g., MADDPG, QMIX) suffer from low sample efficiency and poor convergence in high-dimensional state-action spaces with sparse rewards. (2) Traditional control-based methods (APF, MPC, RRT) lack adaptability to unpredictable obstacle configurations and dynamic target behaviors. (3) Although CL has been introduced into reinforcement learning to improve exploration efficiency and training stability, most existing studies focus on single-agent tasks or relatively simple multi-agent scenarios. For multi-UAV cooperative hunting, especially in environments containing obstacles and a dynamic target, existing curriculum-learning-based MAPPO methods still lack a systematic progressive training mechanism that transfers agents from basic obstacle avoidance to coordinated target encirclement.

To address this gap, this paper proposes a CL-enhanced MAPPO framework, named CL-MAPPO, for multi-UAV cooperative hunting in obstructed environments. The proposed method designs a three-stage curriculum according to the increasing complexity of the hunting task. Curriculum I trains the UAVs to approach a stationary target while avoiding fixed obstacles. Curriculum II introduces randomly generated obstacle layouts and target positions to improve environmental adaptability. Curriculum III further introduces a moving target, requiring the UAVs to learn coordinated pursuit and hunting under dynamic conditions. Through this progressive task decomposition, the UAVs can gradually acquire obstacle avoidance, target searching, formation maintenance, and cooperative hunting capabilities.

Therefore, the main contribution of this study lies in constructing a task-oriented CL strategy for MAPPO-based multi-UAV cooperative hunting, which improves training stability, accelerates convergence, and enhances policy generalization in complex obstacle environments. From an application perspective, the proposed framework is most beneficial for UAV swarm missions that require rapid adaptation in obstacle-rich and uncertain environments, such as cooperative surveillance, border patrol, search-and-rescue target tracking, disaster-area monitoring, and autonomous interception of mobile ground targets. Compared with purely rule-based or direct end-to-end training approaches, CL-MAPPO provides a structured way to acquire reusable skills in a controllable order: first safe obstacle avoidance and target approach, then adaptation to unseen obstacle layouts, and finally dynamic cooperative hunting. These capabilities are important for reducing manual rule design, improving training stability, and enabling UAV teams to form coordinated hunting behaviors under local observations.

### 1.3. Organization

The rest of this paper is organized as follows. Section 2 formulates the multi-UAV cooperative hunting problem in obstructed environments, including the kinematic modeling of UAVs and the construction of the cooperative hunting situation model. Section 3 presents the design of the CL-MAPPO algorithm, encompassing the MAPPO algorithm architecture with the CTDE framework and loss functions, the design of the state space, action space, and composite reward function, and the three-stage CL framework. Section 4 provides extensive simulation experiments, including the configuration of the training environment and hyperparameter settings, ablation experiments to analyze the impact of CL, comparative experiments against MADDPG and MADQN, and qualitative evaluation with trajectory visualization for each curriculum stage and each compared algorithm. Finally, Section 5 concludes the paper and discusses potential directions for future research.

## 2. Problem Formulation and Modeling

### 2.1. Kinematic Modeling for UAV Cooperative Hunting

In practical UAV applications, UAVs naturally operate in three-dimensional space. However, in the cooperative hunting task considered in this paper, the target is a ground target, and all UAVs are assumed to maintain the same flight altitude during the hunting process. Under this assumption, the vertical motion of the UAVs does not directly affect the hunting process. Therefore, the original three-dimensional pursuit problem can be reasonably simplified into a two-dimensional planar motion control problem. This simplification allows the study to focus on the key challenges of cooperative hunting, including target approach, obstacle avoidance, formation coordination, and hunting stability, while reducing the dimensionality of the state and action spaces. In this task, UAVs are required to achieve effective target hunting by maintaining a specific formation configuration. Consequently, it is assumed that each UAV possesses a consistent initial flight heading and that the initial configuration adheres to a standard formation geometry. This paper takes the cooperative hunting of three UAVs as the research object, selects an equilateral triangle as the standard formation configuration, and defines its side length as the target hunting radius.

**Figure 1:**
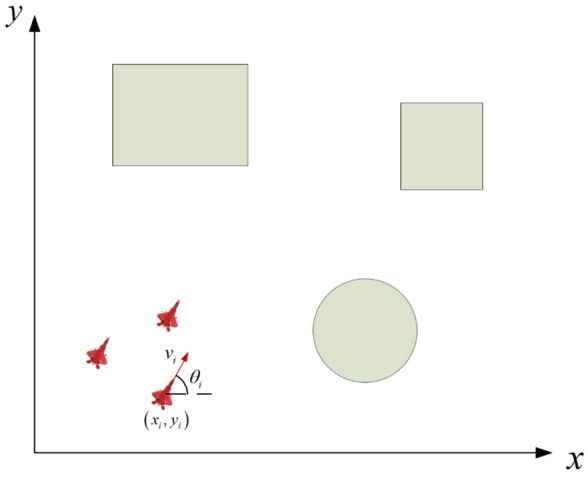
The UAV motion description is in a two-dimensional plane.

A two-dimensional cooperative hunting kinematic model for UAVs is established, in which the state of a UAV comprises its position, velocity, and heading angle, as illustrated in Figure 1.

Let the position of UAV i in the ground inertial coordinate system be denoted as $P_{i}={\left[x_{i},y_{i}\right]}^{T}$, the heading angle $\psi_{i}$ be defined as the angle between the velocity vector and the positive *x*-axis, and the velocity magnitude be $v_{i}$. The kinematic equations of the UAV can be expressed as follows:

(1) $$\left\{\begin{array}{c} {\dot{x}}_{i}=v_{i}\cos\theta_{i}\\ {\dot{y}}_{i}=v_{i}\sin\theta_{i}\\ {\dot{v}}_{i}=a_{v,i}\\ {\dot{\theta }}_{i}=\omega_{i} \end{array}\right.$$

where $a_{v,i}$ denotes the tangential acceleration of the UAV, adjusted through engine thrust to achieve acceleration or deceleration, and $\omega_{i}$ represents the heading angular velocity, achieved through coordinated aileron and rudder maneuvers. UAVs must maintain a certain forward velocity to sustain lift. Hence, a minimum speed constraint $v_{\min}$ exists, and hovering or reverse flight maneuvers are not feasible. Due to aerodynamic constraints, the maximum heading angular velocity is limited by the maximum overload, expressed as $|\omega_{i}|\leq \omega_{\max}$. Limited by engine thrust, the tangential acceleration satisfies $a_{\min}\leq a_{i}\leq a_{\max}$, where $a_{\max}$ represents the maximum acceleration capability and $a_{\min}$ denotes the maximum deceleration capability.

### 2.2. Cooperative Hunting Situation Model Construction

**Figure 2:**
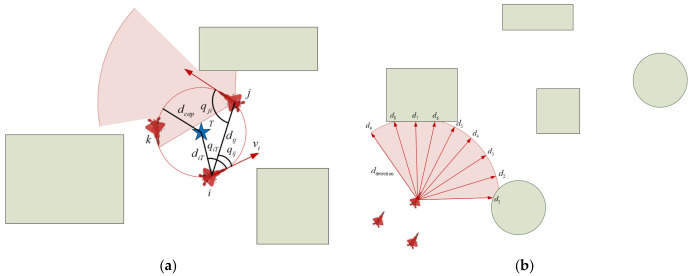
Cooperative hunting situational model in an obstructed environment. (**a**) Hunting model of the UAV. (**b**) Detection model of the UAV (nine radar rays).

The cooperative hunting situation model for UAVs in an obstructed environment is illustrated in Figure 2.

The flight direction vector of the UAV is defined as $v_{i}=[\begin{array}{cc} \cos\psi_{i} & \sin\psi_{i} \end{array}]$. The relative position vectors among UAVs are defined as $d_{ij}=P_{j}-P_{i}$. The relative position vector between UAV *i* and the target is defined as $d_{iT}=P_{T}-P_{i}$.

The transformation relationship between relative situation information and the ground inertial coordinate system is described as follows:

(2) $$\left\{\begin{array}{c} d_{ij}=\left|d_{ij}\right|\\ d_{iT}=\left|d_{iT}\right|\\ \varphi_{ij}=\arccos\left(v_{i}\cdot d_{ij}/d_{ij}\right)\\ \varphi_{iT}=\arccos\left(v_{i}\cdot d_{iT}/d_{iT}\right) \end{array}\right.$$

where $d_{iT}$ denotes the relative distance between UAV *i* and target, $d_{ij}$ denotes the relative distance between UAV *i* and UAV *j*. $\varphi_{ij}$ represents the antenna train angles (ATA) between UAV *i* and UAV *j*, defined as the angle between the velocity vector $v_{i}$ and the relative position vector $d_{ij}$. And $\varphi_{iT}$ represents the ATA between UAV *i* and the target, defined as the angle between the velocity vector $v_{i}$ and the relative position vector $d_{iT}$. The hunting range of the UAV is illustrated in Figure 2a, assuming a maximum hunting distance of $d_{cap}$.

During the cooperative hunting process, each UAV detects its surroundings and acquires distance information relative to obstacles. As illustrated in Figure 2b, the UAV emits nine radar rays within its detection range, spaced at 15° intervals in a plane parallel to the ground. The central ray is aligned with the UAV’s velocity vector $v_{i}$. When no obstacle is present along a given ray, the maximum detection distance $d_{\mathrm{detection}}$ is returned. Let $d_{1}^{i},\;d_{2}^{i},\cdots ,\;d_{9}^{i}$ denote the measured distances along each of the nine ray directions, where $d_{x}^{i}\in \left[0,d_{\mathrm{detection}}\right]$. The normalized obstacle distance information is then defined as $D_{1}^{i},\;D_{2}^{i},\cdots ,\;D_{9}^{i}$, with each component given by:

(3) $$D_{x}^{i}=\frac{d_{x}^{i}}{d_{\mathrm{detection}}},\left(x=1,2\cdots ,9\right)$$

the value of $D_{x}^{i}$ close to 1 indicates that the UAV maintains a safe separation from obstacles in the corresponding direction, whereas $D_{x}^{i}=0$ signifies an imminent collision.

## 3. CL-MAPPO Algorithm Design

### 3.1. Partially Observable Markov Decision Process

The problem of multi- UAV cooperative hunting can be modeled as a POMDP, represented by the tuple (S,O,A,P,Z,R,γ). Where S denotes the global state space, encompassing information such as positions and velocities of all UAVs, ground targets, and obstacles. $O=[o^{1},\ldots ,o^{n}]$ denotes the joint observation space, where $o^{i}$ is the local observation of UAV *i*. $A=[a^{1},\ldots ,a^{n}]$ denotes the joint action space. P:S×A×S→[0,1] is the state transition probability function. Z is the observation function, indicating the probability of receiving observation O after executing action A in state $S^{\prime }$. $R=[r^{1},\ldots ,r^{n}]$ represents the reward function for each agent, and γ∈[0,1] is the discount factor.

At time step t, each UAV selects an action $a_{t}^{i}$ based on its local observation $o_{t}^{i}$ and policy $\pi_{\theta i}(a_{t}^{i}|o_{t}^{i})$, forming a joint action $A_{t}=\left[a_{t}^{i},\ldots ,a_{t}^{n}\right]$. The environment updates to the next state $s_{t+1}$ according to the state transition probability P, and returns an immediate reward $r_{t}^{i}$. The cumulative discounted return for UAV *i* is $R_{i}=\sum_{t=0}^{\infty }\gamma^{t}r_{t}^{i}$. The optimization objective is to find the optimal policy $\pi_{\theta }^{*}$ that maximizes the expected cumulative return.

### 3.2. MAPPO Algorithm

In the task of multi-UAV cooperative hunting of a ground target, UAVs must make decisions based on local observations in dynamic environments while cooperating with other UAVs to form a hunting posture. To address this problem, this section adopts the MAPPO algorithm as the core decision-making framework. The MAPPO algorithm is based on a CTDE architecture, utilizing global information to guide policy optimization during the training phase and relying solely on local observations for decision-making during the execution phase. This effectively addresses the non-stationarity issues prevalent in multi-agent environments.

The MAPPO algorithm is an extension of the Proximal Policy Optimization (PPO) algorithm to the multi-agent domain and adopts an Actor-Critic architecture. In this setup, the Actor network is responsible for generating action policies, while the Critic network evaluates state values. Unlike independent learning PPO algorithms, MAPPO inputs global state information into the Critic network during training, enabling a more accurate assessment of joint action values. During execution, each agent makes decisions independently through its Actor network based solely on local observations.

**Figure 3:**
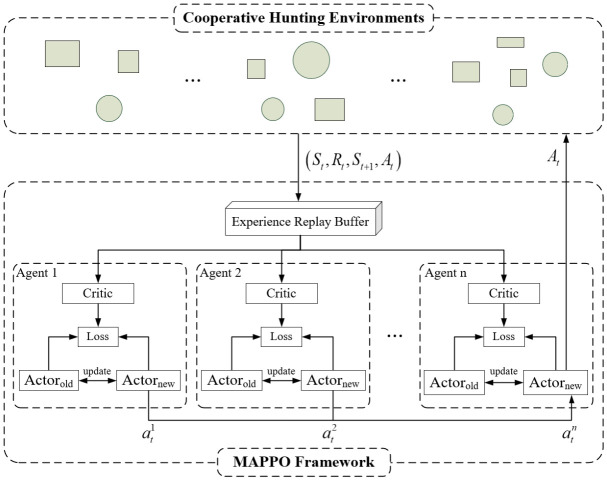
MAPPO-based training framework for cooperative hunting of multiple UAVs.

Figure 3 illustrates the MAPPO-based training framework for cooperative hunting by multiple UAVs. During training, agents output actions based on local observations, the environment provides rewards and updates states, and trajectory data is stored in an experience replay buffer for subsequent network parameter optimization.

The optimization objective of the MAPPO algorithm comprises two components: the policy network loss and the value network loss. The policy network employs a Truncated importance sampling method to ensure stable policy updates while improving sample efficiency.

For agents within the UAVs, the loss function of the policy network is defined as:

(4) $$L^{CLIP}\left(\theta \right)=\frac{1}{BN}\sum_{i=1}^{B}\sum_{k=1}^{N}min\left(r_{\theta ,i}^{\left(k\right)}A_{i}^{\left(k\right)},clip\left(r_{\theta ,i}^{\left(k\right)},1-\varepsilon ,1+\varepsilon \right)A_{i}^{\left(k\right)}\right)$$

where B is the batch size, $r_{\theta ,i}^{\left(k\right)}=\frac{\pi_{\theta }(a_{i}^{\left(k\right)}|o_{i}^{\left(k\right)})}{\pi_{\theta_{\mathrm{old}}}(a_{i}^{\left(k\right)}|o_{i}^{\left(k\right)})}$ is the importance sampling weight, $A_{i}^{\left(k\right)}$ is the estimated advantage function, and ε is the clipping hyperparameter, controlling the maximum step size of policy updates.

The advantage function is computed using Generalized Advantage Estimation (GAE):

(5) $$A_{i}^{\left(k\right)}=\sum_{l=0}^{T-t-1}{\left(\gamma \lambda \right)}^{l}\delta_{t+l}^{\left(k\right)}$$

where $\delta_{t}^{\left(k\right)}=r_{t}^{\left(k\right)}+\gamma V_{\phi }(s_{t+1})-V_{\phi }(s_{t})$ is the temporal-difference error, and λ is the GAE parameter balancing bias and variance.

The loss function of the value network also adopts a clipping approach to prevent excessive value estimation updates:

(6) $$L^{CLIP}\left(\phi \right)=\frac{1}{BN}\sum_{i=1}^{B}\sum_{k=1}^{N}max\left(\begin{array}{l} {\left(V_{\phi }\left(s_{i}^{\left(k\right)}\right)-{\hat{R}}_{i}^{\left(k\right)}\right)}^{2},\\ {\left(clip\left(V_{\phi }\left(s_{i}^{\left(k\right)}\right),V_{\phi old}\left(s_{i}^{\left(k\right)}\right)-\epsilon ,V_{\phi old}\left(s_{i}^{\left(k\right)}\right)+\epsilon \right)-{\hat{R}}_{i}^{\left(k\right)}\right)}^{2} \end{array}\right)$$

where ${\hat{R}}_{i}^{\left(k\right)}=A_{i}^{\left(k\right)}+V_{\phi }(s_{i}^{\left(k\right)})$ is the estimated discounted return.

The total loss function is a weighted sum of the policy loss and value loss, augmented with a policy entropy regularization term to encourage exploration:

(7) $$L\left(\theta ,\phi \right)=L^{CLIP}\left(\theta \right)-c_{1}L^{CLIP}\left(\phi \right)+c_{2}H\left(\pi_{\theta }\right)$$

where $H(\pi_{\theta })$ is the policy entropy, and $c_{1}$, $c_{2}$ are weight coefficients.

The pseudocode for the MAPPO algorithm employed in this study is as follows (Algorithm 1):
**Algorithm 1. The framework of MAPPO**1:Initialize network parameters;2:Initialize experience replay buffer *R*;3:**For** episode *=* 1 to max episode **do**4:**|** Initialize environment, obtain initial global state
$S_{0}$, local observations
$O_{0};$5:**|**  **For** step = 1 to
T **do**
6:****|** **|** For** each UAV *i* **do**7:**|** **|** **|** Select action $a_{t}^{i}$ according to current policy $\pi_{\theta }(\cdot |o_{t}^{i});$8:****|** **|** End For**9:**|** **|** Execute joint action $a_{t}$, reward 
$r_{t}$, next state $s_{t+1}$ observations $o_{t+1}^{i};$
10:**|** **|** Store trajectory data $\left\{s_{t},a_{t},r_{t},s_{t+1}\right\}$ experience replay buffer R;11:**|** **|** Update state $s\leftarrow s_{t+1};$12:****|** End For**13:**|** Sample a batch of data from *R* for training;14:**|** Compute advantage estimates  ${\hat{A}}_{t}^{\left(k\right)}$ and discounted returns ${\hat{R}}_{t}^{\left(k\right)}$ using GAE15:**|** Compute policy gradient and update network parameters;16:**|** Clear replay buffer *R*;17:**End For**18:Save policy network

### 3.3. State Space

The state feature $o^{i}$ of UAV *i* comprises local features $o_{loc}^{i}$, target features $o_{tar}^{i}$ and ally features $o_{ally}^{i}$.

Local features $o_{loc}^{i}=\left\{v_{i},\theta_{i},D_{1}^{i},D_{2}^{i},\cdots ,D_{9}^{i}\right\}$ describe the motion state of UAV *i* itself and the obstacle detection information. It should be noted that a minimum speed constraint $v_{\min}$ applies to UAVs to prevent stalling. Target features $o_{tar}^{i}=\left\{\varphi_{iT},d_{iT}\right\}$ describe the observation information of UAV *i* regarding the target. Ally features $o_{ally}^{i}=\left\{q_{ij},q_{ki},d_{ij},d_{ki}\right\},\left(i\neq j\neq k\right)$ describe the situation information between UAV *i* and allied UAVs.

Consequently, the complete state feature of UAV *i* is represented as $o^{i}=\left\{o_{loc}^{i},o_{tar}^{i},o_{ally}^{i}\right\}$.

### 3.4. Action Space

In the cooperative hunting task, the UAV acts as a continuous motion rigid body system, whose complete dynamic behavior is typically governed by the coupling of multiple aerodynamic control surfaces, such as thrust, elevator, aileron, and rudder. However, to reduce the action dimension and improve policy search efficiency within the reinforcement learning framework, this study simplifies the UAV control input to a two-dimensional continuous action vector $\left[a_{v,i},\omega_{i}\right]$. Where $a_{v,i}$ represents the tangential acceleration command along the longitudinal axis of UAV *i*, and $\omega_{i}$ denotes the yaw rate command about the vertical axis.

### 3.5. Reward Function

The reward function is designed to guide the UAVs to complete the cooperative hunting task in a progressive and stable manner. Specifically, the distance reward encourages each UAV to approach the target. The angle reward guides the UAVs to form an approximately equilateral hunting structure around the target. The obstacle avoidance reward penalizes unsafe proximity to obstacles. The formation consistency reward maintains reasonable distances among UAVs. The success reward provides a large positive signal when the target is successfully captured, and the step penalty encourages the UAVs to complete the task efficiently.

The composite reward is therefore not only used to maximize the hunting success rate, but also to balance multiple task requirements, including safety, cooperation, formation stability, and efficiency. This reward design is consistent with the progressive curriculum structure, allowing the UAVs to first learn basic obstacle avoidance and target approach behaviors, and then gradually acquire more complex cooperative hunting strategies.

1. Distance reward

The distance reward is designed to encourage UAVs to continuously approach the target. Let the distance between UAV *i* and the target at time *t* be $d_{iT,t}$, and the hunting radius be $d_{\mathrm{cap}}$. The distance reward $r_{dist,i}$ is defined as:

(8) $$r_{dist,i}=\left\{\begin{array}{ll} d_{iT,t-1}-d_{iT,t}, & d_{iT,t}>d_{\mathrm{cap}}\\ 1, & d_{iT,t}\leq d_{\mathrm{cap}} \end{array}\right.$$

this design encourages UAVs to prioritize approach speed at long distances while focusing on maintaining position once within the hunting radius.

2. Angle and formation reward

In order to achieve a uniform hunting of the target, the three UAVs are expected to maintain an angular separation of approximately $120^{{}^{\circ}}$ relative to the target. Accordingly, an angle reward $r_{\mathrm{ang}}$ is introduced, which is derived from the interior angles of the triangle formed by the current positions $P_{1},P_{2},P_{3}$ of the three UAVs:

(9) $$r_{\mathrm{ang},i}=1-\frac{\left|∠P_{1}-60^{{}^{\circ}}\right|+\left|∠P_{2}-60^{{}^{\circ}}\right|+\left|∠P_{3}-60^{{}^{\circ}}\right|}{180^{{}^{\circ}}}$$

where the reward function measures the deviation of each interior angle from $60^{{}^{\circ}}$, thereby encouraging the UAVs to form a near-equilateral triangular formation around the target, which corresponds to an ideal symmetric encirclement.

Furthermore, to prevent formation failure due to excessive or insufficient spacing between UAVs, a formation reward $r_{\mathrm{form},i}$ is introduced:

(10) $$r_{\mathrm{form},i}=-I\{d_{near}>1.25d_{\mathrm{cap}}\}-I\{d_{near}<0.25d_{\mathrm{cap}}\}$$

where $d_{near}$ is the distance between UAV *i* and its adjacent UAVs, I⋅ is the indicator function. This reward encourages UAVs to maintain reasonable separation distances.

3. Obstacle avoidance reward

UAVs must avoid obstacles and remain within boundaries. The environment detects surrounding obstacles via 9 rays, returning a normalized distance vector $D_{x}^{i}\in [0,1]\left(x=1,\ldots ,9\right)$. The obstacle avoidance reward $r_{obs,i}$ consists of detection distance, reward and collision penalty.

The detection distance reward encourages UAVs to stay away from obstacles, with obstacles located in front of the UAV incurring a greater penalty than those to the sides:

(11) $$r_{detect,i}=-\exp\left(-\left(\sum_{x=1}^{9}D_{x}^{i}\cos\left(\alpha_{x}\right)/\sum_{x=1}^{9}\cos\left(\alpha_{x}\right)\right)\right)$$

where $\alpha_{x}$ denotes the angle between the x-th radar ray and the primary heading direction of the UAV.

A collision penalty is applied upon collision with an obstacle:

(12) $$r_{collision,i}=\left\{\begin{array}{ll} \;-100, & if\;\;collision\;occurs\\ \;0 & else \end{array}\right.$$

The composite obstacle avoidance reward is $r_{obs,i}=r_{detect,i}+r_{collision,i}$.

4. Success reward and step penalty

Hunting is considered successful when the following conditions are simultaneously met for all three UAVs: the distance between each UAV and the target is less than the hunting radius $d_{\mathrm{cap}}$, the target is located inside the triangle formed by the three UAVs, and the three UAVs have circled the target for three complete orbits. Upon successful triggering, a substantial one-time reward $r_{success}=300$ is granted, and the current episode terminates. Additionally, to encourage rapid task completion, a minor time penalty is applied at each step:

(13) $$r_{\mathrm{step}}=-0.05\cdot \Delta t$$

where Δt is the simulation step size.

5. Comprehensive reward

The comprehensive reward $r_{i,t}$ for the UAV *i* is defined as the following linear combination:

(14) $$r_{i,t}=\left(0.25\cdot r_{dist,i}+0.25\cdot r_{\mathrm{ang},i}+0.25\cdot r_{\mathrm{form},i}+0.25\cdot r_{obs,i}\right)\cdot \Delta t+r_{success}+r_{\mathrm{step}}$$

where weights balance objectives, including target approach, obstacle avoidance, formation keeping, and successful task completion. This reward function provides dense and effective learning signals for the multi-agent reinforcement learning algorithm, guiding the UAV swarm to collaboratively accomplish the hunting task in complex, obstructed environments.

### 3.6. CL Framework

**Figure 4:**
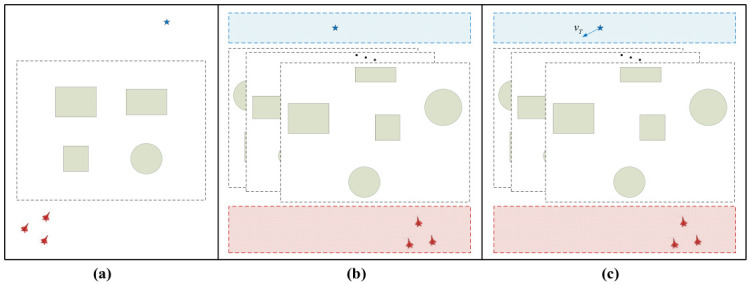
Three-stage CL environment settings. (**a**) Curriculum I: fixed obstacles and a stationary target. (**b**) Curriculum II: random obstacle positions and counts, random target position. (**c**) Curriculum III: adds random target movement.

In complex cooperative hunting tasks, the high dimensionality of the state-action space and the highly dynamic nature of the environment often lead to prolonged training cycles, convergence difficulties, and suboptimal policy performance during direct training. To address this, a CL strategy based on progressive environmental difficulty is designed in this section, comprising three stages, as shown in Figure 4.

Figure 4a illustrates the environment setup for Curriculum I. This stage employs fixed spatial distributions of obstacles to simulate simplified mountainous or canyon terrain. The target position is fixed, and the hunting UAVs are initialized randomly within a small range. The training objective is for UAVs to master fundamental obstacle avoidance, target search, and hunting capabilities, laying the groundwork for subsequent complex tasks.

Figure 4b illustrates the environment setup for Curriculum II. In this stage, the positions and quantities of obstacles are randomly generated to simulate more variable mountainous or canyon terrains. The target position is randomized, and the hunting UAVs are initialized randomly over a larger area. This setup aims to enhance the UAVs’ adaptability and decision-making capabilities for hunting in complex, obstructed environments.

Figure 4c illustrates the environment setup for Curriculum III. Building upon Curriculum II, this stage further increases task difficulty by endowing the target with simple random mobility. The target velocity is assumed to be half the maximum flight speed of the UAVs. Obstacle distribution and UAV initial positions remain randomized. This environment compels the agents to learn effective hunting strategies against dynamic targets while simultaneously managing complex static obstacles, thereby enhancing their task execution capabilities in realistic scenarios.

**Figure 5:**
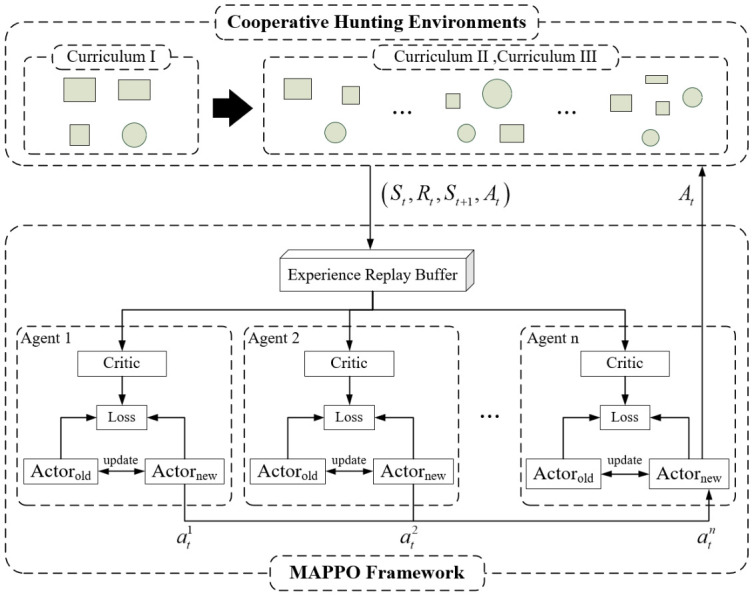
CL-MAPPO-based decision model for cooperative hunting in an obstructed environment.

This study integrates CL with MAPPO to propose the CL-MAPPO algorithm. Figure 5 illustrates the UAV cooperative hunting decision-making model in obstructed environments based on the CL-MAPPO algorithm. The operational flow of the model is as follows: The Actor network generates actions for the UAVs, which interact with the environment based on these actions. The resulting transition $\left\{S_{t},A_{t},R,S_{t+1}\right\}$ is stored in an experience replay buffer for subsequent network training. During training, the maneuvering strategy acquired from a given curriculum serves as the initial policy for the subsequent curriculum. Upon completing the training across all curricula, a high-quality cooperative hunting policy is ultimately obtained. The overall training workflow of the CL-MAPPO algorithm for multi-agent cooperative hunting decision-making is depicted in Figure 5.

## 4. Experimental Design and Results Analysis

### 4.1. Simulation Environment and Parameter Settings

The environmental settings for Curriculum I, Curriculum II, and Curriculum III for the cooperative hunting task are presented in Table 1, where $\left(x_{1},y_{1},v_{1},\theta_{1}\right)$ denotes the coordinates, velocity, and heading angle of the UAV initially positioned closest to the target. Throughout the training process, the UAV’s minimum speed $v_{\min}$ is set to 50, the maximum speed $v_{\max}$ is set to 150, the hunting radius $d_{cap}$ is 300, and the maximum detection distance $d_{\mathrm{detection}}$ is 800.

The number of training episodes for each curriculum was set to 2000. This choice was based on empirical observations from preliminary experiments: in Curriculum I, the UAVs began to obtain successful hunting experiences at approximately 1000 episodes, after which the cumulative reward steadily increased and converged around 2000 episodes. Therefore, 2000 episodes per stage ensures sufficient convergence while maintaining reasonable training cost. Adaptive or performance-based curriculum transitions (e.g., triggering the next curriculum after a sustained reward threshold is reached) are promising directions for future work. In this study, a fixed-episode transition strategy is adopted to ensure experimental consistency and comparability.

The algorithm was implemented in Python 3.9, and all training and simulation experiments were conducted on a computer equipped with an Intel(R) Core(TM) i9-14900KF CPU and an NVIDIA GeForce RTX 4070 GPU. The hyperparameter settings for the algorithm are listed in Table 2. To ensure a fair comparison, CL-MAPPO and the baseline algorithms are trained under the same network architecture and basic training settings whenever applicable. The discount factor, learning rates, optimizer and hidden layer size are kept consistent across different experimental groups.

### 4.2. Ablation Experiment

**Figure 6:**
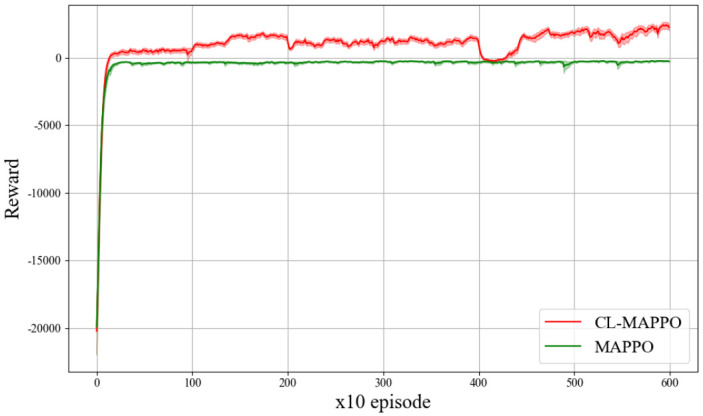
Reward convergence curves for CL-MAPPO and MAPPO algorithms.

To investigate the effectiveness of CL, an ablation experiment was conducted by comparing the proposed CL-MAPPO algorithm with the baseline MAPPO without CL. As shown in Figure 6, both CL-MAPPO and MAPPO were trained for 6000 episodes. Since CL only changes the task difficulty progression without modifying any training hyperparameters, the training time is approximately the same for both algorithms. The faster convergence of CL-MAPPO compared to vanilla MAPPO can be attributed to the curriculum-based task decomposition. In Curriculum I, the task difficulty (fixed obstacles, stationary target) is substantially lower than the original full task. This allows the agents to quickly learn fundamental skills such as obstacle avoidance and target approach, without being overwhelmed by simultaneous challenges. The policy acquired in this easy stage provides a warm start for the next curriculum, effectively reducing the initial exploration burden. Consequently, CL-MAPPO reaches higher reward levels earlier than MAPPO, which must solve the full complex task from scratch. Notably, a temporary reward drop occurs at each curriculum transition (e.g., from Curriculum I to II). This phenomenon is expected and instructive. The policy learned in Curriculum I is tailored to a simpler environment (fixed obstacles, stationary target). When the same policy is applied to Curriculum II (random obstacles/positions, dynamic target), its performance degrades because the new task demands greater environmental adaptability. The subsequent recovery of reward indicates that the agents are able to adapt the previously learned skills to the more challenging setting. This demonstrates that while the policy is not directly transferable without loss, it serves as a valuable initial point that accelerates re-convergence compared to learning from a random policy. Thus, within the same training time, incorporating CL enables CL-MAPPO to achieve faster convergence, higher cumulative rewards, and a more robust final hunting policy than MAPPO without CL.

### 4.3. Comparative Experiments

**Figure 7:**
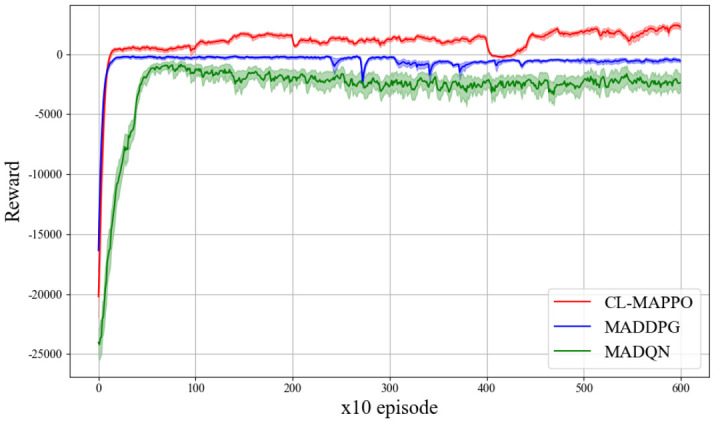
Reward convergence curves for different algorithms (CL-MAPPO, MADDPG, MADQN).

To further evaluate the effectiveness of the proposed algorithm, comparative experiments were conducted among CL-MAPPO, MADDPG, and MADQN. The cumulative reward per episode during training for each algorithm is shown in Figure 7.

As illustrated by the reward convergence curves in Figure 7, the CL-MAPPO algorithm maintains the highest cumulative reward level throughout the training phase. Moreover, the reward value steadily increases and ultimately converges to a significantly positive range as training progresses. This result indicates that the CL-MAPPO algorithm can learn an effective and stable cooperative hunting strategy, successfully achieving the task target. In contrast, under the same task environment, the cumulative rewards for the MADDPG and MADQN algorithms consistently remain below zero until convergence, suggesting that the strategies learned by these algorithms in complex environments are virtually incapable of successfully capturing the target. Specifically, the CL-MAPPO algorithm benefits from a phased progressive training mechanism based on CL. Although each curriculum transition is accompanied by a temporary drop in reward, CL-MAPPO can rapidly re-converge based on the prior guidance from CL, ultimately acquiring a generalizable policy for handling complex dynamic scenarios. Conversely, due to the lack of a phased guidance mechanism, the MADDPG and MADQN algorithms directly confront highly complex task environments from the initial stage, making their policies prone to becoming trapped in local optima or stagnation, with post-convergence rewards remaining negative. This stark contrast underscores the significant advantage of the CL strategy in enhancing the convergence efficiency and final decision-making performance of multi-agent reinforcement learning for cooperative hunting tasks.

**Figure 8:**
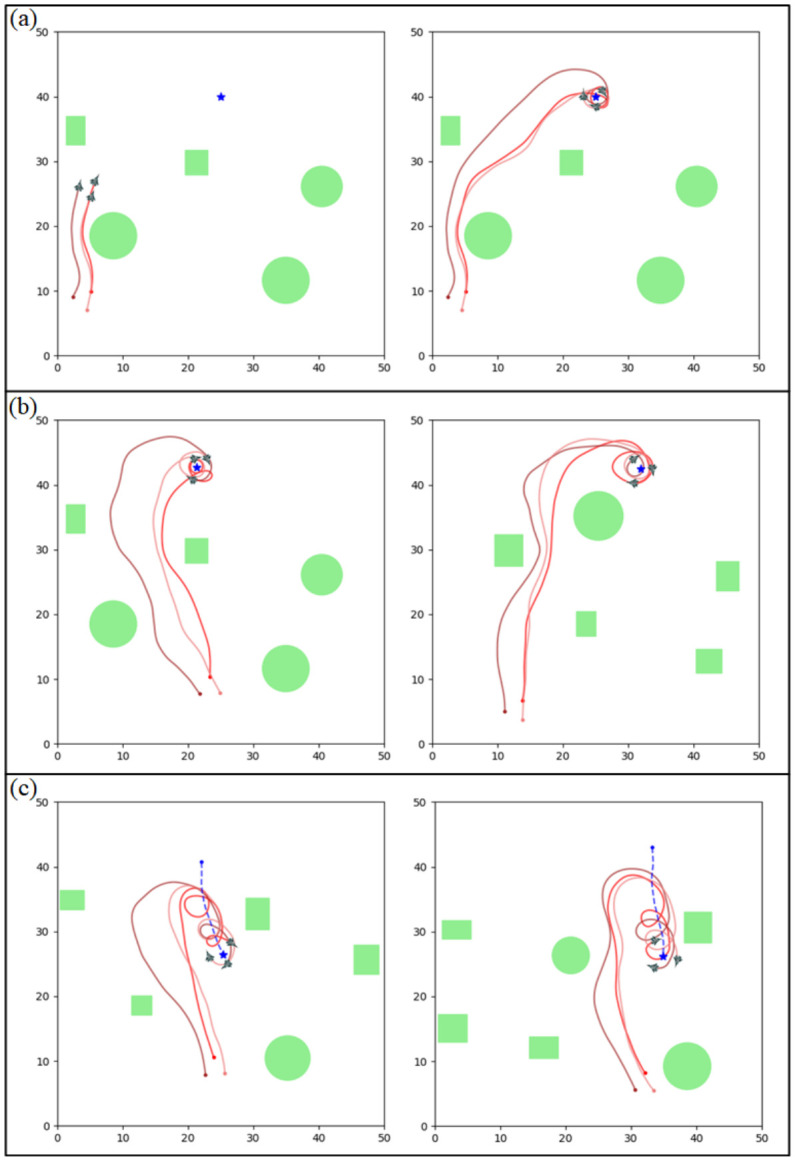
Trajectory visualization of UAV hunting performance after each curriculum stage. (**a**) Curriculum I. (**b**) Curriculum II. (**c**) Curriculum III.

To further validate the actual hunting performance of the trained policies, the network models saved upon completion of each curriculum stage were selected for qualitative evaluation and trajectory visualization in the test environment, with results shown in Figure 8.

Figure 8a presents the hunting performance after training in Curriculum I. At this stage, the UAV formation can stably maintain its configuration in a fixed obstacle environment, effectively avoid obstacles, and perform search and hunting tasks against a stationary target. The policy acquired in this stage possesses fundamental behavioral capabilities such as obstacle avoidance flight, target search, and formation encirclement.

During the Curriculum II training phase, the environment features complex and variable obstacle distributions, with target positions initialized randomly. The trajectories shown in Figure 8b demonstrate that the UAV formation can flexibly adapt to diverse terrains and obstacle layouts, safely and accurately completing the hunting task for random fixed targets while maintaining a relatively intact formation structure. Training at this stage significantly enhances the decision-making adaptability of the UAV policy in complex, obstructed environments.

Curriculum III training further introduces random maneuvering capabilities of the target within the complex obstacle environment. As shown in Figure 8c, the UAV formation can maintain its configuration, continuously tracking and encircling the moving target, and safely completing the hunting task across various complex, obstructed environments. Observing the specific morphology of the hunting trajectories, the UAV formation tends to approach the hunting circle from the target’s flank, subsequently forming a loitering posture around the target to complete the hunting. This phenomenon correlates with the maneuvering characteristics of the UAVs employed. In summary, through the three-stage progressive curriculum training, the resulting UAV hunting policy exhibits the capability to handle complex obstacle scenarios and efficiently hunt dynamic maneuvering targets, thereby validating the effectiveness and practicality of the CL framework in complex tasks.

The obstacle configurations in curriculum III are randomly generated at the beginning of each episode. Specifically, both the positions and the number of obstacles vary across episodes. The UAVs have no prior knowledge of the obstacle layout and can only perceive nearby obstacles through their onboard radar observations. Therefore, each episode presents a previously unseen environment to the UAVs.

This randomization mechanism continuously exposes the agents to different obstacle layouts and obstacle densities during training and evaluation. As a result, the learned policy is not limited to a fixed obstacle map, but is trained to adapt to diverse and unseen obstacle configurations. The experimental results show that CL-MAPPO can maintain stable hunting behavior under such randomized environments, demonstrating its generalization ability in obstacle-rich scenarios.

**Figure 9:**
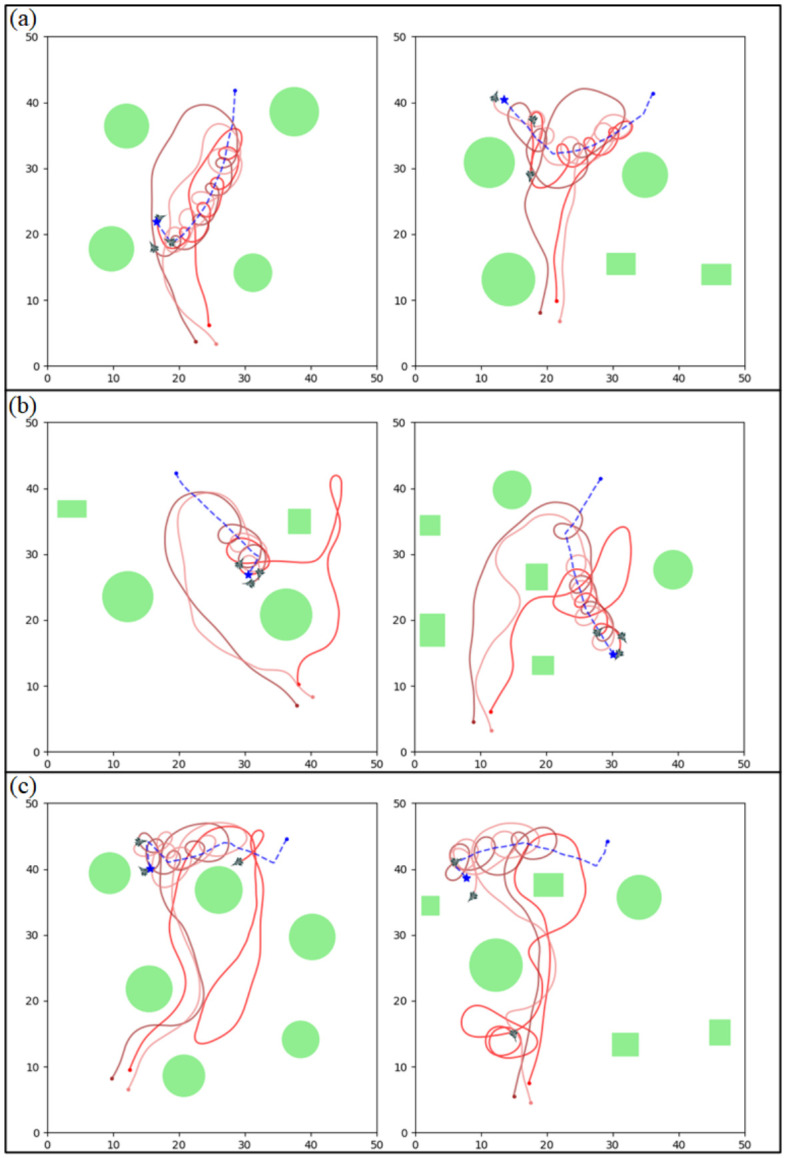
Trajectory visualization of baseline algorithms. (**a**) MAPPO. (**b**) MADDPG. (**c**) MADQN.

The network models saved upon completion of training for MAPPO, MADQN, and MADDPG were selected for qualitative evaluation and trajectory visualization, with results shown in Figure 9.

As shown in Figure 9a, the policy learned by MAPPO enables the UAV formation to maintain a relatively stable configuration and safely approach the target while performing hunting maneuvers. The three UAVs exhibit a coordinated converging trend toward the target region, indicating that the formation possesses basic cooperative hunting awareness. However, the hunting effectiveness remains insufficient, as the target can easily escape from the encircling circle before a tight hunting is established. The overall hunting intention is relatively weak. Specifically, the UAVs exhibit overly conservative obstacle avoidance behavior, abandoning the hunting attempt even when a considerable distance remains from the obstacles. This excessive caution can easily prevent the formation from closing in on the target effectively, resulting in hunting failure.

Figure 9b shows that the MADDPG policy allows the UAV formation to accomplish obstacle avoidance and attempt target encirclement. The UAVs demonstrate a general tendency to move toward the target while navigating around obstacles. However, the algorithm’s overall cooperative hunting awareness is relatively weak, and it completely fails to maintain the formation configuration at the initial hunting stage, with each UAV approaching the target independently, causing the desired triangular formation structure to collapse. Although basic target approach and obstacle avoidance capabilities are present, it is difficult to achieve effective cooperative hunting.

As illustrated in Figure 9c, the MADQN algorithm exhibits the worst performance among the three compared algorithms. The learned policy only enables basic obstacle avoidance with only a weak formation maintenance capability at the initial hunting stage. Moreover, only two agents approach the target and attempt encirclement, while the third agent easily abandons its intention to encircle, moves independently, and plays a weak role throughout the engagement, showing no discernible cooperative pattern. This indicates a relatively poor policy learning outcome for cooperative hunting.

**Figure 10:**
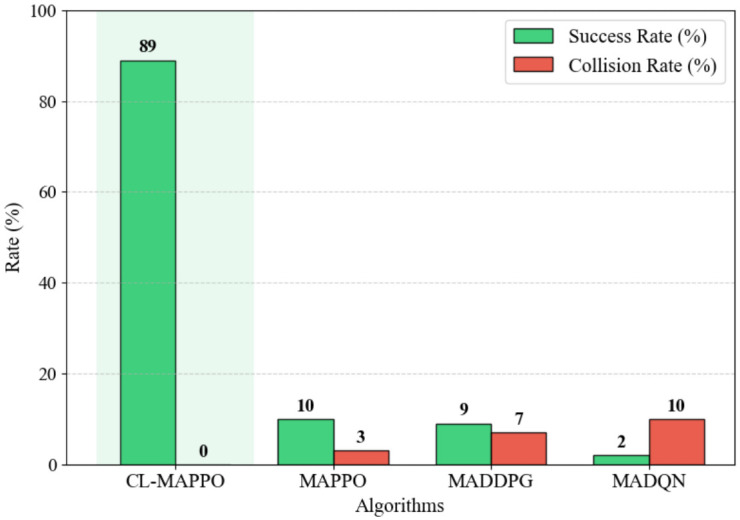
Algorithm performance comparison in terms of success rate and collision rate for CL-MAPPO, MAPPO, MADDPG, and MADQN.

Figure 10 presents a quantitative comparison of four algorithms, namely CL-MAPPO, MAPPO, MADDPG, and MADQN, in terms of success rate and collision rate over 100 hunting episodes. CL-MAPPO achieves the highest success rate (89%) and the lowest collision rate (0%). Through a three-stage curriculum learning, the UAV formation acquires robust capabilities in formation keeping, obstacle avoidance, target search, and cooperative hunting. MAPPO yields a success rate of 10% and a collision rate of 3%. Its policy is overly cautious, abandoning hunting attempts too early and allowing the target to escape easily. MADDPG shows a success rate of 9% and a collision rate of 7%. Cooperative hunting awareness is weak, and the triangular formation collapses at the initial stage, impairing effective encirclement. MADQN performs worst, with a success rate of 2% and the highest collision rate of 10%. The learned policy enables only primitive obstacle avoidance and minimal cooperation, with one agent often leaving the hunting attempt.

In summary, CL-MAPPO significantly outperforms the other three algorithms. The curriculum learning framework effectively balances safety and hunting aggressiveness, overcoming the limitations of over-conservatism (MAPPO), formation collapse (MADDPG), and poor cooperation (MADQN) in complex multi-UAV hunting tasks. These results highlight the practical advantage of the proposed three-stage curriculum. The UAVs do not need to learn obstacle avoidance, formation keeping, and moving-target hunting simultaneously from the beginning. Instead, each stage provides a more learnable subproblem and transfers the acquired policy to the next stage. As a result, the final policy demonstrates capabilities that are difficult for the baseline algorithms to obtain directly in the complete task, including safer navigation in cluttered spaces, more stable triangular formation maintenance, and persistent pursuit of a maneuvering target. Such capabilities are particularly valuable for time-critical autonomous swarm missions in which manual rule tuning is difficult, and the environment cannot be fully predefined.

## 5. Conclusions

This paper proposed a curriculum learning-based multi-agent proximal policy optimization algorithm, termed CL-MAPPO, for multi-UAV cooperative hunting in obstructed environments. A three-stage progressive curriculum was designed to decompose the complex hunting task. Curriculum I uses fixed obstacles with a stationary target. Curriculum II introduces random obstacles and target positions. Curriculum III further incorporates a dynamic maneuvering target. Experimental results demonstrate the effectiveness of the proposed approach, which effectively addresses the key challenges of low exploration efficiency, slow environmental adaptation, and difficult convergence in multi-agent cooperative hunting tasks. The proposed CL-MAPPO framework provides a practical and scalable solution for UAV swarm operations in obstacle-rich and uncertain environments.

Beyond target intelligence, two additional limitations should be acknowledged [30]. First, the current study considers only three UAVs in a two-dimensional planar environment. While the 2D simplification is valid for ground-target hunting at constant altitude, scaling the approach to larger swarms (e.g., 5 or more UAVs) may introduce new challenges. Nevertheless, the CTDE architecture of MAPPO is known to scale reasonably well, and we expect that increasing the number of agents would be feasible with moderate modifications to the reward structure and network capacity. Second, full three-dimensional flight dynamics (e.g., altitude changes, vertical obstacle avoidance) are not considered. Extending the current framework to 3D would require reformulating the kinematic model, updating the obstacle detection rays to a spherical grid, and redesigning the formation reward. These extensions are left as important directions for future research.

## Figures and Tables

**Table 1:** Cooperative hunting environment settings.

Symbol	Curriculum I	Curriculum II	Curriculum III
$x_{1}$	500	$1000\leq x_{1}\leq 4000$	
$y_{1}$	900	$500\leq y_{1}\leq 1000$	
$x_{T}$	4000	$1000\leq x_{T}\leq 4000$	
$y_{T}$	4000	$4000\leq y_{T}\leq 4500$	
$\theta_{1}$	π/4	$\left[\pi /4,3\pi /4\right]$	
$v_{T}$	0		$v_{\max}/2$
$v_{1}$	100		

**Table 2:** Hyperparameter settings.

Hyperparameter	Symbol	Value
Discount factor	*γ*	0.99
Number of hidden layers	/	2
Hidden layer size	/	128
Optimizer	/	Adam
Policy network learning rate	/	$1\times 10^{-4}$
Value network learning rate	/	$1\times 10^{-3}$
Batch size	Bs	20
Rollout episodes	/	15
Clipping parameter	ε	0.2

## Data Availability

The data presented in this study are available upon request from the corresponding author.

## Abbreviations

The following abbreviations are used in this manuscript:

PPO	Proximal policy optimization
MAPPO	Multi-agent proximal policy optimization
CL	Curriculum learning
UAV	Unmanned aerial vehicle
DRL	Deep reinforcement learning
APF	Artificial potential field
MADRL	Multi-agent deep reinforcement learning
VDN	Value-decomposition networks
MADDPG	Multi-agent deep deterministic policy gradient
TD3	Twin delayed deep deterministic policy gradient
CTDE	Centralized training decentralized execution
